# Oral traditional Chinese patent medicines combined with non-steroidal anti-inflammatory drugs for primary dysmenorrhea: A protocol for Bayesian network meta-analysis and systematic review

**DOI:** 10.1371/journal.pone.0276129

**Published:** 2022-10-21

**Authors:** Zhe Chen, Yingying Peng, Xiaoyu Qiang, Geliang Song, Fengwen Yang, Bo Pang, Hui Wang

**Affiliations:** 1 Evidence-based Medicine Center, Tianjin University of Traditional Chinese Medicine, Tianjin, China; 2 First Teaching Hospital of Tianjin University of Traditional Chinese Medicine, Tianjin, China; 3 National Clinical Research Center for Chinese Medicine Acupuncture and Moxibustion, Tianjin, China; PhD, PLOS, UNITED KINGDOM

## Abstract

**Introduction:**

Primary dysmenorrhea (PD) was the most common gynecological disorder, with an increasingly high prevalence worldwide. PD often accompanied other dysmenorrhea-associated symptoms to trigger exacerbations, and even cause depression and anxiety for patients. As the effective first-line medication, non-steroidal anti-inflammatory drugs (NSAIDs) have become widespread across China and combined with oral traditional Chinese patent medicines (TCPMs) for PD in clinical practice. We hope to provide better efficacy and safety evidence about oral TCPMs combined with NSAIDs (oral TCPMs+NSAIDs) for patients with PD by this network meta-analysis.

**Methods and analysis:**

We will perform a Bayesian network meta-analysis of all oral TCPMs+NSAIDs for clinical diagnosis as PD. PubMed, Embase, Cochrane Library, Web of Science, China National Knowledge Infrastructure, Wanfang Data Knowledge Service Platform, VIP information resource integration service platform databases, and clinical registers will be searched from the database inception to June 30, 2022 to find randomized controlled trials. Two reviewers will independently screen and check titles and abstracts and read the full text. Data extraction with the same criteria will be conducted by two researchers, including study characteristics, participant characteristics, interventions and comparators, and outcomes. We will perform the network meta-analysis by the Bayesian random method to analyze the direct and indirect comparisons. Meta-regression with multiple covariates will be conducted to find the potential heterogeneity. We will perform the sensitivity analysis to identify the potential effect on the robustness of our results. Evidence certainty of all interventions in outcomes will be identified and assessed by Grading of Recommendations, Assessment, Development, and Evaluation (GRADE) assessment. Funnel plots with Egger test and Begg’s test to detect the potential publication bias.

**Trial registration:**

PROSPERO registration number: CRD42021265675.

## Introduction

Primary dysmenorrhea (PD), as the common gynecological disorder among women, has been broadly defined as painful menstruation without underlying identifiable pelvic pathology [1]. Different definitions and assessment methods have been used to evaluate the prevalence of PD, which affected between 45 and 95% of women and reported the high prevalence rate in different regions around the globe [2]. Compared to the prevalence of PD in different colleges through multiple studies, results revealed that the incidence among female students even ranges up to 89.1% [2, 3].

Painful menses or cramps in the hypogastrium were the major clinical features of PD. Many patients also have been jeopardized by some accompanying dysmenorrhea-associated symptoms such as headache, gastrointestinal reactions (nausea and vomiting), genitourinary symptoms, weakness, flushing, anxiety, and others [4, 5]. As a leading risk factor for fibromyalgia, PD will seriously affect the patient’s quality of life, mood, and sleep quality if painful menses and cramps cannot be effectively controlled by various therapeutic applications [2, 6]. The clinical manifestations of dysmenorrhea were significantly related to menarche, menstrual duration, and menstrual flow [4, 7]. In addition, relevant research has demonstrated that PD has been seriously affected and induced by psychological burden, life pressure, and family environment, which have been considered higher risk factors of PD exacerbation [3, 8].

Most PD patients have used non-steroidal anti-inflammatory drugs (NSAIDs) as the first-line therapy and most effective medication with a well empiric response and high evidence quality of recommendation to relieve pain throughout the day [9, 10]. NSAIDs have commonly been indicted for women with PD during menstruation, including ibuprofen, naproxen sodium, mefenamic acid, Celecoxib, etc [4, 11]. The long-term use and a high frequency of NSAIDs in unsupervised conditions have failed to relieve painful menses through multiple pathological mechanisms and generate NSAID-related adverse reactions [12]. Flurbiprofen and tiaprofenic acid may be the optimal treatments for PD according to the results of Network Meta-Analysis [13]. However, with the emergence of NSAID-resistant dysmenorrhea and NSAID-related side effects among individual NSAIDs, people also tend to become more cautious about the NSAIDs for PD [12, 14].

Due to the cognitive differences in the management of menstrual pain, self-care education has been needed for dysmenorrhea and decreased the misuse of NSAIDs and other pharmaceutical methods [15]. In China, when clinical doctors recommend using traditional Chinese medicine (TCM) to treat PD, they often emphasize and urge patients and their families to pay attention to self-care and management at any of the phases of the medication use process. TCM with the recommendation base on the evidence has been considered possibly efficient for PD [16]. Therefore, more and more researchers have conducted more in-depth research for PD with TCM.

In the short-term treatment process, patients with PD had better responses and satisfactory safety to TCM therapy, which may be correlated to the regulation of the MAPK pathway in the underlying mechanisms [17, 18]. TCM can effectively relieve pain and PD’s other related symptoms by improving endocrine and metabolic disorders and controlling the expression of core genes related to PD [19]. Oral traditional Chinese patent medicines (TCPMs), as the national TCM preparations containing various herbal materials, have been using for clinical practice and were even known as the specific drugs for gynecological disease (PD, amenorrhea, infertility, etc) in China [20, 21]. TCPMs, including various TCMs, as the TCM products, according to the prescribed prescription and preparation process and approved by the National Medical Products Administration in China, to prevent and treat diseases [22]. Various oral TCPMs for menstrual diseases, have also been currently cataloged in the Chinese pharmacopoeia and approved for production and registration by the national medical products administration. Using selective and rapid UHPLC-MS/MS methods to detect the oral TCPMs, relevant data confirmed that a plethora of potential chemical markers of TCPMs has analgesic, spasmolysis regulation, and cardiovascular protective effects to achieve the anti-dysmenorrhea effect [23, 24]. In addition, TCPMs also have the good curative effect on improving the PD’s pain, and its mechanism may be related to regulating the prostaglandin in uterine tissue and inhibiting uterine contraction [25, 26].

Based on the combination of oral TCPMs and NSAIDs in China, we will compare and analysis the pain relief with primary concern, related indicators, and safety of this combination to select the best interventions that oral TCPMs combined with NSAIDs (TCPMs+NSAIDs) in the clinic. We will provide better safe and efficient treatment regimens about oral TCPMs+NSAIDs for patients with PD by this network meta-analysis, to solve the current dilemma in clinical treatment.

## Methods and analysis

### Protocol and registration

We registered the protocol of this network meta-analysis on the International Prospective Register of Systematic Reviews (PROSPERO): CRD42021265675. This study will be conducted and reported according to the Preferred Reporting Items for Systematic Reviews and Meta-Analyses (PRISMA) checklist, PRISMA protocol statement, and the PRISMA-extension statement for network meta-analysis [27–29].

### Eligibility criteria

We comprehensively review and consider the eligibility criteria of PICOS (populations, interventions, comparators, outcomes, and study designs) in all potential studies, the steps of which will be described in more detail below. If necessary, we will report and explain the significant modifications of this protocol by the final published results of this network meta-analysis.

#### Study designs

We will include the randomized controlled trials (RCTs) without any limitations of quality methods in blinding and allocation concealment. Since the purpose of the study, single-arm non-randomized trial and cross-over trial are not meet the eligibility criteria. In addition, case reports, cohort studies, case-control studies, and cross-sectional studies that did not meet our inclusion criteria will be excluded, as well as some journal papers, degree papers, and conference abstracts with missing data. Although oral TCPMs were widely applied in China, included studies will be not restricted by language and geographical locations to make it more comprehensive for our network meta-analysis.

#### Populations

Our target populations will be reproductive age women with PD according to the guidelines and expert consensus without serious complications (multiple organ failure, exacerbation of acute infectious diseases, etc.) and other conditions (pregnant women, lactating women, severe malnutrition due to gynecological diseases, etc.). Patients with PD have been diagnosed with the patient’s self-reported, pelvic examination, ultrasound, and laparoscopy, and there is no obvious pelvic pathology. The influence of living environment, dietary habits, and drug dependency can cause abnormal menstruation (early or delayed menstruation), which may aggravate the symptoms of PD. To ensure the stability of the disease, avoid the influence of secondary dysmenorrhea and minimize the potential influencing factors, so we set the study population on women of reproductive age. In addition, we will stratify by baseline age in the data processing stage to ensure a comprehensive interpretation of the results if possible. If patients’ age did not meet our criteria, accompanied by serious complications, and diagnosed as secondary dysmenorrhea caused by pelvic pathology, they will be excluded from the study. We do not limit the race, educational region, country, duration of the disease, and the severity of PD to ensure that more studies will be included to support the results. Patients with drug resistance to NSAIDs should also be excluded.

#### Interventions

The interventions of the treatment group are oral TCPMs+NSAIDs to treat PD. The purpose and modalities of the sudden emergency medication strategy must be unaffected by the studied drugs for this network meta-analysis. Oral TCPMs should be registered on the website of the national medical products administration and completely reported the detailed information of the company and batch number in included studies. Usage and dosage of NSAIDs in the treatment group will be consistent with the comparators. Other Chinese medicine therapies (TCM decoction, hospital preparations, acupuncture, massage, etc.) compared with NSAIDs or the placebo will be excluded.

#### Comparators

For the control group, the comparators will be the NSAIDs or placebo. Although we do not limit the types of NSAIDs, the clinical dosage and usage have to be carried out by clinicians according to the guidelines. There are numerous kinds of NSAIDs in the clinic as the first-line treatment for PD, including indomethacin, naproxen, ibuprofen, diclofenac, meloxicam, celecoxib, etoricoxib, parecoxib, etc. Beyond this, NSAIDs as routine treatment and routine nursing will be included in our study. Single or combined use of hormones, oral contraceptives, and other non-NSAIDs does not meet the inclusion criteria.

### Outcomes

#### Primary outcomes

We choose the pain intensity by visual analogue scale (VAS) and pain duration as the Primary outcomes in our network meta-analysis based on the PD’s clinical therapeutic purposes. VAS was widely used as the principal method to measure subjective pain for patients with PD. By presenting for patients a horizontal line and two extremes with relevant fixed values, respondents can choose the most appropriate point to represent the pain score according to their perception of pain (wewers1990). Pain grade that before and after treatment for patients is evaluated by the VAS with a range from 0–10 cm. To better reflect the objectivity of our results, pain durations that from the beginning to the relief of pain are selected as another primary outcome based on the VAS or patients’ self-report.

#### Secondary outcomes

Secondary outcomes include response rate, Cox Menstrual Symptom Scale (CMSS), quality of life (QOL), and Safety (adverse events). We will calculate the response rate as [(number of total patients − number of invalid patients)/number of total patients] × 100%.

### Search strategy

We take the comprehensive literature search that follows the professional search strategy with keywords (free words combine with subject words) to find RCTs, and the keywords include “oral traditional Chinese patent medicine”, “non-steroidal anti-inflammatory drugs”, “primary dysmenorrhea”, dysmenorrhea, “randomized controlled trials”, and so on. The detailed search strategy of this study will be shown in Table 1. Due to oral TCPMs have a long application history in China, we set the retrieval time from the database inception to June 30, 2022 to make the retrieval strategy more comprehensive and the detailed information as follows:

1. Database retrieval: PubMed, Embase, Cochrane Library, Web of Science, China National Knowledge Infrastructure (CNKI), Wanfang Data Knowledge Service Platform, and VIP information resource integration service platform databases will be searched by two researchers to find published peer-reviewed journal papers or potentially unpublished papers according to the same inclusion criteria. We also included unpublished studies and grey literature in this network meta-analysis and systematic review.
2. Manual retrieval: The potentially relevant articles which met the eligibility criteria and reference lists of related systematic reviews and included studies will be checked and searched by manual retrieval to supplement the unsearched paper or unpublished grey literature. We will also search the clinical trial registry to find potential studies.
3. Contact with author and journal: we will try to contact the author or journal for missing data or clarification for unclear information by e-mail or other means.

**Table 1:** Search strategy.

**### Searching of Primary Dysmenorrhea**
#1 “Primary Dysmenorrhea” [MeSH]
#2 Dysmenorrhea [MeSH]
#3 “Menstruation Disturbances” [MeSH]
#4 (“primary dysmenorrhea” OR dysmenorrhea OR “menstruation disturbances” OR “menstrual colic” OR “painful menstruation” OR menalgia OR algomenorrhea OR “menstruation disorders” OR “menstrual disorder” OR “pelvic pain” OR “painful period” OR “period pain” OR “menstrual pain” OR cramps) [TIAB]
#5 = #1 OR #2 OR #3 OR #4
**### Searching of Oral Traditional Chinese Patent Medicine**
#6 “Oral Traditional Chinese Patent Medicine” [MeSH]
#7 “Traditional Chinese Patent Medicine” [MeSH]
#8 “Traditional Chinese Medicine” [MeSH]
#9 (“traditional Chinese medicine” OR “traditional Chinese patent medicine” OR “oral traditional Chinese patent medicine” OR “Aifu Nuangong Pills” OR “Danggui Shaoyao Granules” OR “Wuji Baifeng Pills” OR “Dan’e Fukangjian” OR “Tongjingbao Granules” OR “Tongjingling Granules” OR “Yuanhu Zhitong Tablets” OR “Tongjing Oral Liquid” OR “Tongjingning Granules” OR “Tongjingning Syrup” OR “Guizhi Fuling Capsules” OR “Shuerjing Granules” OR “Shuerjing Capsules” OR “Bawei Tongjing Tablets” OR “Tianqi Tongjing Capsules” OR “Tianqi Tongjing San” OR “Funv Tongjing Pills” OR “Funv Tongjing Granules” OR “Fuke Tiaojing Pills” OR “Fuke Tiaojing Granules” OR “Tiaojing Huoxue Capsules” OR “Yuanhu Zhitong Oral Liquid” OR “Yuanhu Zhitong Soft Capsules” OR “Sanjie Zhengtong Capsules” OR “Qizhi Xiangfu Pills” OR “Vinegar Xiangfu Pills” OR “Shixiao San” OR “Tongjing Ointment” OR “Dingkun Dan” OR “Fuke Tiaojing Capsules” OR “Shaofu Zhuyu Capsules” OR “Shaofu Zhuyu Pills” OR “Fuke Tiaojing Tablets” OR “Tiaojing Huoxue Tablets” OR “Zhitong Huazheng Capsules” OR “Wenjing Pills” OR “Yuanhu Zhitong Dropping Pills” OR “Yuanhu Zhitong Dispersible Tablets” OR “Yuanhu Zhitong Capsules” OR “Yuanhu Zhitong Granules”) [TIAB]
#10 = #6 OR #7 OR #8 OR #9
**### Searching of Non-Steroidal Anti-inflammatory Drugs**
#11 “Non-Steroidal Anti-inflammatory Drugs” [MeSH]
#12 (“Non-Steroidal Anti-inflammatory Drugs” OR “NSAIDs” OR “aspirin” OR “tiaprofenic acid” OR “valdecoxib” OR “diclofenac” OR “fketoprofen” OR “mefenamic” OR “nimesulide” OR “piroxicam” OR “lurbiprofen” OR “mefenamic acid” OR “ibuprofen” OR “indomethacin” OR “rofecoxib”) [TIAB]
#13 = #11 OR #12
**### Combined with the Drugs**
#14 = #10 AND #13
**### Searching of Study Design**
#15 “Randomized Controlled Trials” [Publication Type]
#16 “Randomized Controlled Trials” [MeSH]
#17 (“randomized controlled trial” OR “randomized controlled study” OR “randomized trial” OR “randomized study” OR “randomized placebo-controlled study” OR “randomized parallel-group study” OR “controlled clinical trial” OR “multicenter study” OR “double-blinded controlled study”) [TIAB]
#18 = #15 OR #16 OR#17
**### ALL**
**#19 = #5 AND #14 AND #18**

### Literature screening and data extraction

#### Study selection

**Fig 1:**
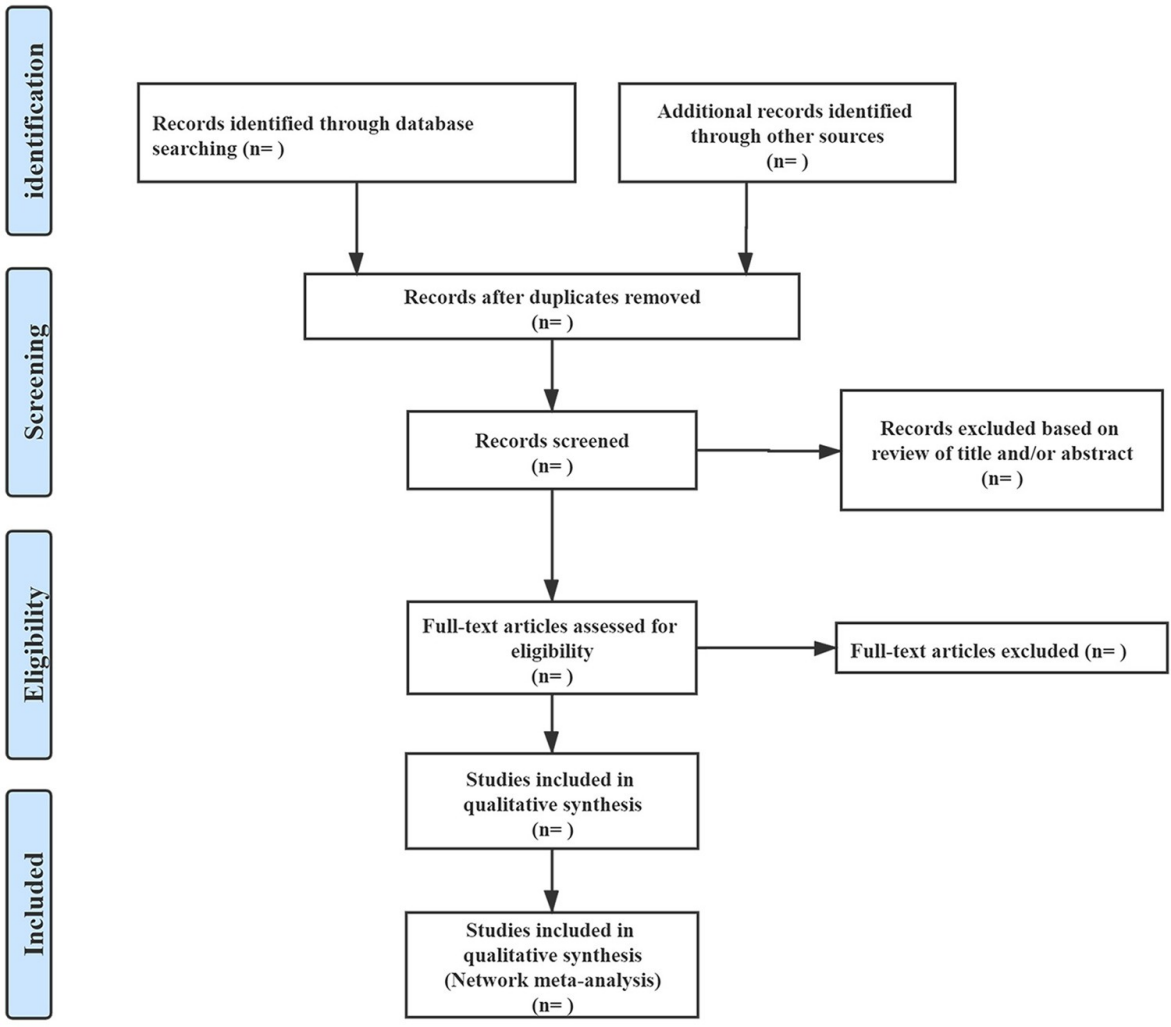
Summary of evidence search and selection.

Two researchers (ZC and YP) will independently screen the publications from the retrieval strategy in this network meta-analysis by cross-process, which will greatly guarantee the accuracy and reliability of data. Removing duplicated studies by the check of all retrieved literature, remaining articles will be evaluated. Firstly, the titles and abstracts of the remaining potential eligible articles will be screen. Then they read the full text and screen the baseline characteristics and important data information to make a confirmed decision. In literature screening and data extraction, any problems will be discussed and negotiated by two researchers. A third researcher will resolve disagreements by consensus, if necessary. The flow diagram of study selection (Fig 1) will be generated according to PRISMA, including records identified through database searching, additional records identified through other sources, records after duplicates removed, records screened based on the review of title and/or abstract, full-text articles assessed for eligibility, and studies included in the qualitative synthesis. Removing studies will be recorded and explain the reasons for the exclusion.

#### Data extraction

The data extraction will be conducted independently and checked by two researchers (ZC and YP) according to the same criteria, which save in their data collection tables. If there are any differences or unclear data, it is necessary to discuss with senior professionals to make consistent results. Lack of data and vague data expression and definition need to communicate with authors and journals and obtain accurate data information as much as possible. All data must be cross-checked again before data processing and data analysis. The extracted data and information are as follows:

1. Study characteristics: author, publication year, study type and design, country, funding, recruitment duration, sampling approach, and others.
2. Participant characteristics: gender must be female, race, age, sample size, number of patients lost to follow-up, course of treatment, course of disease, follow-up time.
3. Interventions and comparators: detailed implementation process in the treatment and control group, interventions and comparators information included brand name, generic name, dosage and usage, manufacturer and batch number, and duration and route of administration.
4. Information of outcomes: The values of pain intensity, CMSS, and QOL will be reported before and after treatment. Pain duration, response rate, and adverse events only report the values after the completion of trials. The original study provides the value of the summary estimate, which will compare with the original data to verify consistency.

### Risk of bias assessment

All included RCTs will be evaluated by two researchers (XQ and GS) independently with the same assessment criteria according to the Cochrane risk of bias tool [30]. Two researchers will assess and discuss the five quality assessment items with the same criterion for each included study in this systematic review. Detailed information of the risk of bias items as follow: Bias arising from the randomisation process, bias due to deviations from intended interventions, bias due to missing outcome data, bias in measurement of the outcome, and bias in selection of the reported result. The judgment of each item will include the three grades (low, some concerns, and high risk of bias). The third researcher (HW) will discuss and resolve the disagreement and obtain the unified standard by consultation. We used the Cochrane risk of bias 2.0 tool to assess the risk of bias, including summary and each included study.

### Statistical analysis

#### Data analysis

We will calculate binary outcomes as odds ratios (ORs) with the corresponding 95% credible interval (CrI) and continuous outcomes as mean differences (MDs) with the corresponding 95% CrI, respectively. MDs and log (OR) values will be derived from the posterior distribution of the model that reported the medians with their corresponding 95% CrI. We will perform the network analysis by the Bayesian random method to analyze the direct and indirect comparisons of all interventions in all studies. The heterogeneity of between-studies will be set under a vague with prior conditions for all means, and assumed a common variance (τ) with a uniform prior distribution for all comparisons between studies. We will choose the optimized Markov chain Monte Carlo (MCMC) method, which runs four chains with weighted sample size. The iterations will be set as least 200,000 to obtain model convergence after an initial burn-in of 50 000 and a thinning of 10, which is assessed using the Brooks–Gelman–Rubin method with a potential scale reduction factor up to 1. The ranking of interventions will be evaluated by the surface under the cumulative ranking curve (SUCRA) for each outcome, further to find the best SUCRA to support our results. Because the included studies may contain indirect and direct comparations, we will perform the node splitting method to calculate the inconsistent evidence differences between all comparations in this systematic review. We used the R statistical software (version 4.0.5; gemtc, RJAGS, BUGSnet, and meta packages) for data analysis, including the network plots, forest plots, and funnel plots [31–34].

#### Assessment of heterogeneity

When P<0.1, there is significant statistical heterogeneity in each outcome; there is statistical heterogeneity not significant (P≥0.1). According to the I^2^ value of statistical heterogeneity, we will choose the different effect models. The heterogeneity will be divided into three degrees with different ranges of estimated I^2^ values: low heterogeneity (under 25%), moderate heterogeneity (25%-50%), and high heterogeneity (over 50%) [35].

#### Meta-regression

Univariate meta-regression with multiple covariates will be conducted to find the sources of potential heterogeneity, which may affect the stability of our network meta-analysis results and decrease the potential modifiers. We will select the potential factors that are most likely to affect the stability of outcome indicators and the scope of application of outcomes as the important covariates according to the research purposes, clinical concerns, and patients’ characteristics. Multiple covariates may include the types of NSAIDs, sample size, course of the disease, time of intervention, gender, age, living environment, dietary habits, return visits, disease prognosis, etc.

#### Sensitivity analysis

Sensitivity analysis will be performed to identify the potential effect on the robustness of results in the different conditions. We will visually compare the results of some possible factors and exclude the other non-relative factors to assess our results. We will verify the robustness and reliability of our results by identifying and dealing with the traits that are most likely to affect the results from many factors (such as types of NSAIDs, sample size, age, quality publication, etc).

#### GRADE assessment of certainty of evidence

To identify the evidence certainty of all interventions, using the Grading of Recommendations, Assessment, Development, and Evaluation (GRADE) framework to assess the cumulative evidence follow the seven aspects (downgraded because of risk of bias, inconsistency, indirectness, imprecision, publication bias, intransitivity, and incoherence). The GRADE assessment has four grades of evidence certainty for each intervention, including high, moderate, low, and very low certainty [36, 37].

#### Assessment of publication bias

Each outcome that included at least 10 studies will be conducted the funnel plots by R 4.0.5 to test and assess the potential publication bias as per Cochrane guidelines in the pairwise comparisons. Egger test and Begg’s test will be used to detect the asymmetry distributions in outcomes.

### Patient and public involvement

This network meta-analysis will be conducted to assess and prove the published RCTs retrieved from multiple source databases. There is no patient or public involvement in our study, so patients and the public will not be involved in the progress of the research question, conception, study design, evaluation, and discussion.

### Ethics and dissemination

Due to we will conduct the network meta-analysis to systematic review based on the secondary literature, ethics and dissemination do not apply to this study. The results of this systematic review will be published after peer-review.

## Discussion

The compelling clinical evidence and widely accepted therapies of oral TCPMs are still lacking in the treatment of PD. Thus, oral TCPMs+NSAIDs will be systematically reviewed by synthesizing the existing literature. This network meta-analysis aimed to demonstrate the best efficacy and safety evidence of oral TCPMs+NSAIDs for PD firstly. We will explore and report the clinical efficacy and safety of oral TCPMs+NSAIDs according to the results of pain intensity, pain duration, related secondary outcomes, and adverse events. Univariate meta-regression will be conducted to explore and detect multiple modifiers, which can ensure the stability of this network meta-analysis and reduce the impact of heterogeneity in our results. In addition, we will also use sensitivity analysis to enhance the reliability and robustness of the results in the types of NSAIDs, sample size, age, and quality publication. Finally, considering the potential benefits and hazards and the value of SUCRA, we will recommend the optimal interventions of oral TCPMs+NSAIDs to patients with PD in this network meta-analysis. The quality of included studies and the potential risk of publication bias may affect the results of this systematic review. The strengths and limitations will be discussed in detail.

Previous systematic reviews found that TCM provides therapeutic advantages for PD [16], and this systematic review will provide high-quality evidence for oral TCPMs+NSAIDs in the treatment of PD in clinical applications. In addition to reducing pain and improving the quality of life of patients with PD, the results from this network meta-analysis will also provide a certain reference value for clinical decision-making and guidelines. Definitive evidence of oral TCPMs+NSAIDs, as the supplement of TCM in the treatment of PD, can be beneficial in terms of TCM clinical treatment and provide some insights for other gynecological diseases associated with PD. The efficacy of TCM can provide a certain theoretical basis and advantages for treating PD [38], and our results will provide evidence to enhance the health of PD patients and an outlook on the development direction of future treatment.

This comprehensive and rigorous network meta-analysis will provide the assessment of the current evidence of oral TCPMs+NSAIDs for PD. Based on the results of the study, we will publish this network meta-analysis in a peer-reviewed journal.

## Supporting information

S1 File PRISMA-P (Preferred Reporting Items for Systematic Review and Meta-Analysis Protocols) 2015 checklist: Recommended items to address in a systematic review protocol have been found in support material. (DOC)
